# Metal–organic framework gels and monoliths

**DOI:** 10.1039/c9sc04961d

**Published:** 2019-11-14

**Authors:** Jingwei Hou, Adam F. Sapnik, Thomas D. Bennett

**Affiliations:** a Department of Materials Science & Metallurgy , University of Cambridge , 27 Charles Babbage Road , Cambridge , CB3 0FS , UK . Email: tdb35@cam.ac.uk

## Abstract

The synthesis of metal–organic frameworks (MOFs) has, to date, largely been in the form of crystalline powders. However, interest in different physical morphologies of this class of materials is growing. In this perspective, we provide an overview of the structure, properties and applications of MOF monoliths. In particular, we explore the complex synthetic landscapes associated with MOF crystallization and discuss the synthetic factors leading to the formation of MOF gels, *i.e.* the precursor to sol–gel MOF monoliths. Finally, we provide our thoughts on the future development of this field, and attempt to highlight the importance of the MOF gel state in the discovery of new functional materials.

## Abstract 2

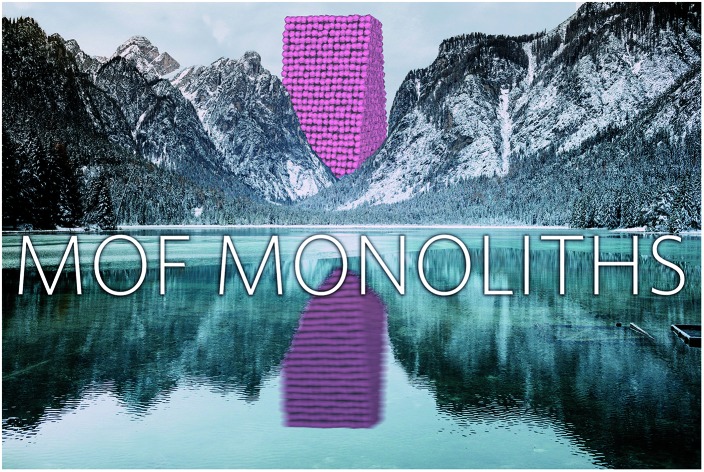
This perspective links the synthesis of MOF Gels to the formation of MOF-monoliths and their resultant properties and application.

## Introduction

Metal–organic frameworks (MOFs) are obtained through the self-assembly of metal nodes with organic ligands, and represent a huge emerging class of functional materials. They are considered potential solutions for numerous environmental and energy challenges, including access to clean water, air pollution reduction and renewable energy supplies.^1^–^4^ Interest in the family is primarily because design, modification and alteration of their chemical structures are possible *via* the use of a host of different chemical building units, regulation of synthetic parameters or utilization of post-synthetic reaction chemistry. These changes result in different chemical and structural properties, which impact upon potential applications in separation, adsorption, catalysis and gas storage.^5^–^9^

**Fig. 1:**
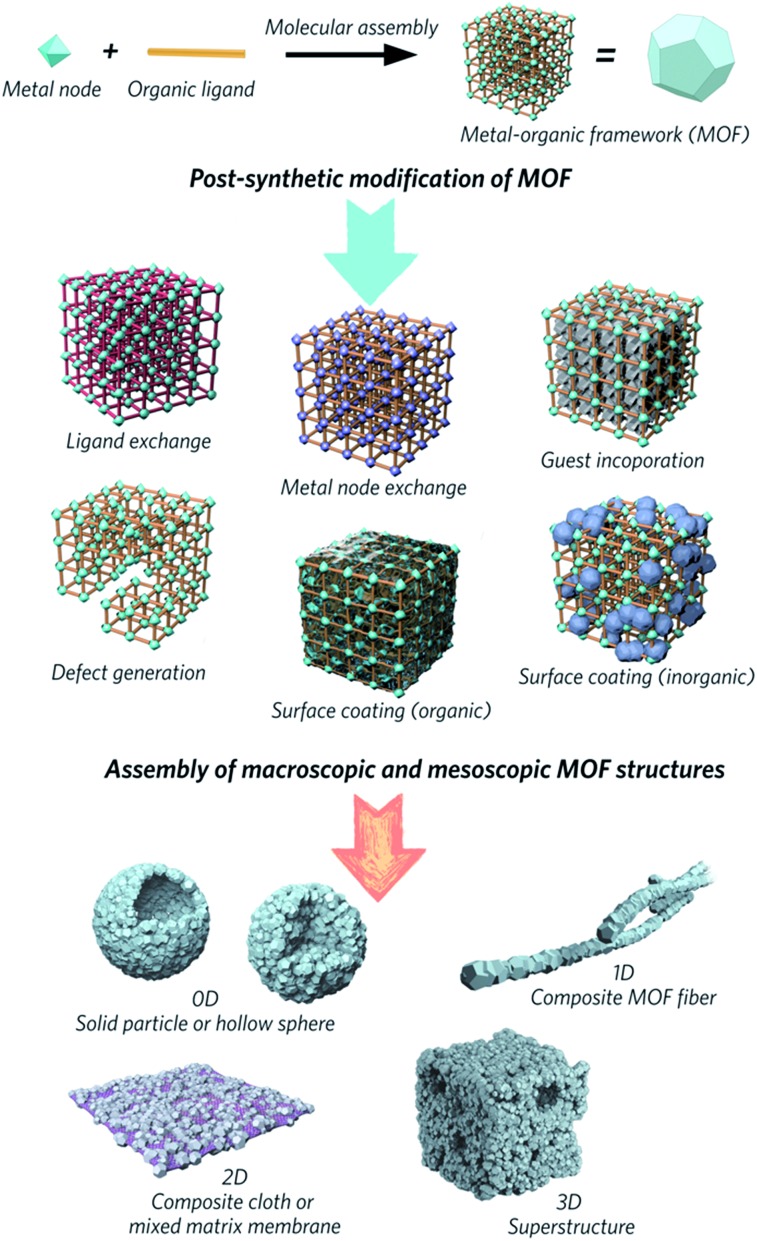
Schematic overview of post-synthetic modifications and meso/macroscopic morphological assemblies of MOFs.

Attention has slowly been shifting from the chemical structure and properties of microcrystalline MOF powders, to the relationship between physical morphology, structure and applications of these interesting materials (Fig. 1). The majority of MOFs are synthesized as polydisperse microcrystalline powders. These may suffer from inherent problems such as poor handling properties, mass transfer limitations, and mechanical instability.^10^,^11^ For example, the “free” powder form of a MOF packed within an adsorption column can lead to a significant pressure drop over time, caused by the gradual compaction of the powder with pressure, resulting in higher mass resistance within the column.^12^ In the area of catalysis, the use of MOFs in powder form usually results in difficulty recycling the catalyst.^13^,^14^ Importantly, while the search for high surface area MOFs is given great gravitas, it is gravimetric and volumetric gas adsorption capacities that are of higher importance for solid-state storage systems in industry.^15^,^16^

The United States Department of Energy (DOE) 2015 technical target for an on-board hydrogen storage system is given as 0.09 kg H_2_ kg^–1^, or 0.081 kg H_2_ L^–1^, respectively.^17^ Large quantities of void space between individual discrete crystallites in powders thus reduces the packing density and therefore compromises volumetric adsorption capacities.^18^,^19^ A bottleneck for the use of MOFs is therefore the assembly of practical devices with a high powder packing density, or in structurally coherent, robust, continuous morphologies.^20^

The fabrication of bulk architectures has been approached in a number of ways,^21^ including the use of binders, mechanical compression, or through the blending of active MOF components in polymeric substrates. These mixed-matrix membranes,^22^,^23^ for example, offer enhanced processabilities and mechanical stabilities compared to purely crystalline powders, though effective loadings are limited due to reduced colloidal stabilities during the fabrication process. Blocking of the internal porosity of the MOF component is also possible, and reduces the accessibility of the micropores to guest molecules.^24^ Methodologies for shaping MOFs without the use of a secondary component, and producing “pure” bulk MOF materials are therefore highly sought after.

The formation of sol–gel-derived MOF monoliths, in particular, has come to prominent attention,^12^,^25^,^26^ due to the ability to structure different pore size regimes within the same material, and the increased capabilities of volumetric uptakes of gas. However, the formation mechanism of these bulk architectures is poorly understood, despite several publications showing that their existence is linked to the prior formation of a MOF gel state.

The gel state is formally defined by IUPAC as a “non-fluid colloidal network or polymer network that is expanded throughout its whole structure by a fluid”.^27^ The MOF gel state has been shown, by several publications, to be a colloidal network of discrete crystalline nanoparticles that aggregate *via* weak non-covalent interactions throughout a liquid phase. For clarity, the MOF gel state is a subset of coordination polymer gels,^28^*i.e.* a type of metal–organic gel (MOG), that is comprised solely of discrete nanoparticles of crystalline MOF. Hence, with smaller particle sizes favouring the formation of MOF gels, the propensity to form a MOF gel is itself linked to the factors behind the crystallization of MOFs.

An important aim in this field is the ability to generalize monolith formation. To do so, one must first generalize MOF gel formation, where the avoidance of microcrystalline powder products is a pre-requisite. An understanding of the crystallization process is therefore essential in order to obtain particles sizes which favour the formation of MOF gels.

This perspective aims to summarize the state-of-the-art work on the fabrication of MOF-based monolithic architectures, and concentrates on linking the formation of sol–gel MOF monoliths to the mechanistic aspects of MOF gel formation. This focus clearly delineates from the excellent summary of the wider area of sol–gel processing of MOFs by Falcaro *et al.*^29^ Finally, we present open questions for this area, and hypothesize that many of the 70 000 known MOF structures will be accessible in the monolithic state.^30^

## Mechanistic aspects of MOF gel formation

Formation of the MOF gel state, *i.e.* the precursor to MOF monoliths, has been linked to the supramolecular aggregation of MOF nanoparticles. These may be monodisperse, though this is not necessary. This can be achieved by first understanding, and subsequently controlling, the crystallization process. This not only determines the periodic structure of the MOF, but also the crystallite size and distribution. Hence, controlling crystallization processes facilitates the targeted synthesis of monodisperse MOF nanoparticles.

### Crystallization of MOFs

The self-assembly of MOFs can be understood in terms of classical crystallization, which consists of two processes: nucleation and crystal growth.^31^,^32^ Classical crystallization typically occurs from a supersaturated solution. Once supersaturation has been reached, nucleation can begin to occur. As nuclei are introduced into the system crystal growth starts to occur. Often, both processes occur in competition with each other, both depleting the concentration of reactants in the solution. Once the concentration has decreased sufficiently, both processes will terminate. The rates of nucleation and crystal growth can be quantified using the Avrami and Gualtieri models.^31^,^33^ In addition to the rates, the Avrami exponent, *n*, can be extracted from crystallization data. This gives an indication of the mechanism and dimensionality of the crystal growth.^31^,^34^–^36^ An *n* > 1 indicates the crystal growth is phase-boundary-limited, *i.e.* it occurs through successive monomer-attachment on the surface of the particles. An *n* = 4 suggests a monomer-attachment process occurring in three dimensions alongside continuous nucleation. On the other hand, *n* < 1 suggests a diffusion-limited mechanism of growth.^37^ These models go some way to providing a mechanistic way to rationalise the size and distribution of crystallites in terms of kinetic parameters. In practice, it is a delicate balance between the relative rates of nucleation and crystal growth that dictates the crystallite size and dispersity. For example, a comparatively fast nucleation rate with respect to crystal growth results in the formation of small, monodisperse, crystallites. This is well understood in terms of the LaMer “burst” mechanism.^38^ Hence, understanding how the rates of nucleation and crystal growth are affected by synthetic parameters can allow for targeted syntheses of monodisperse crystalline nanoparticles.

Despite the use of relatively simple models to understand crystallization, it is nonetheless a complex phenomenon and even in the simplest cases is not fully understood. For example, Yeung *et al.* recently highlighted disparities in the literature surrounding the crystallization of a prototypical MOF, ZIF-8 (Zn(mIm)_2_, where mIm = 2-methylimidazolate).^39^ Specifically, *in situ* powder X-ray diffraction (PXRD), pH and turbidity measurements were used to show that the rate of crystallization decreased with increasing reactant concentration. A pre-equilibrium model consisting of metastable intermediate clusters was proposed in order to rationalise their somewhat counterintuitive findings. Within this framework they could explain how increasing reaction concentration inhibits both nucleation and crystal growth. Further analysis showed that the kinetics of crystallization also varied as a function of reaction progress. Such a dynamic mechanism for a seemingly simple system indicates the level of complexity associated with the crystallization of MOFs.

### MOF gel formation

**Fig. 2:**
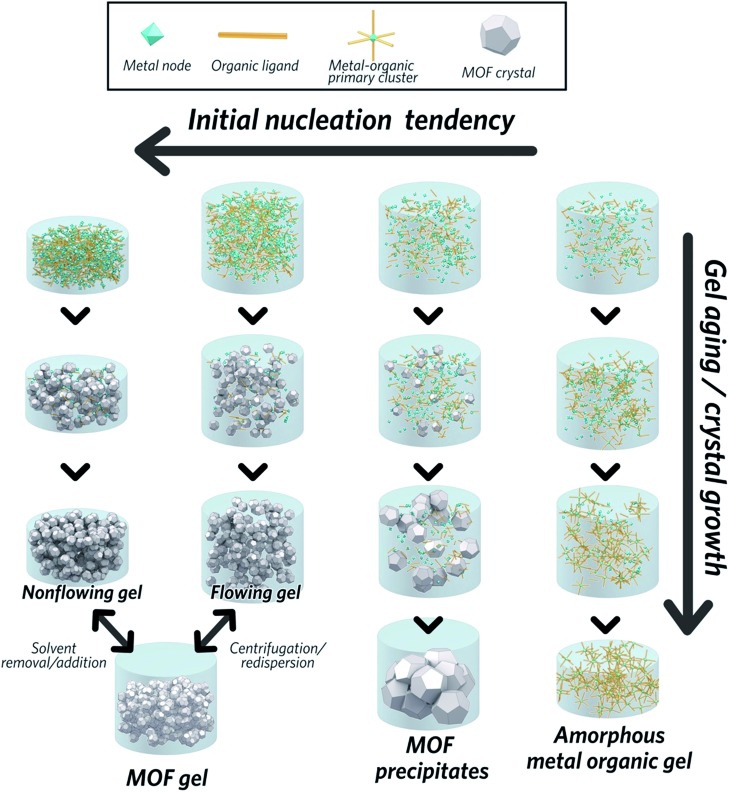
Mechanism of the formation of a MOF gel, precipitate and metal–organic gel.

The formation of MOF gels is based on colloidal chemistry, beginning with the generation of crystalline MOF nanoparticles with a narrow size distribution. Weak non-covalent interactions between nanocrystals may then compete with continued crystal growth, maximising the potential for gelation (Fig. 2).

Key examples of gel formation of several prototypical MOFs; ZIF-8,^40^ MIL-53 (Al(OH)BDC, where BDC = benzene-1,4-dicarboxylate),^41^ HKUST-1 (Cu_3_(BTC)_2_, where BTC = benzene-1,3,5-tricarboxylate)^12^ and several Zr-based frameworks,^42^ have recently been reported. Such cases are often represented by the “test-tube inversion test”, *i.e.* inverting a test tube and using the non-flowing nature to identify the gel state. This is not a rigorous test, however, and can lead to the misidentification of viscous liquids as gels.^43^

In work on the gelation properties of several Zr-based MOFs with different ligands, including both pristine and functionalized UiO-66 (Zr_6_O_4_(OH)_4_(BDC)_6_), UiO-67 (biphenyl dicarboxylate), MOF-801 (fumarate), MOF-808 (benzene-1,3,5-tricarboxylate) and NU-1000 (1,3,6,8-tetrakis(*p*-benzoate)pyrene), Bennett *et al.* proposed gelation to be a consequence of rapid formation of a high concentration of nanocrystals in solution. This promptly consumes the reactants within the precursor solution, impeding further growth to form microcrystalline MOF species and subsequent precipitation. As the viscosity increases, the nanocrystals gradually aggregate to form a gel state, with a weakly, non-covalently bonded colloidal network structure throughout the liquid volume, that adopts the shape of the container. The non-covalent interactions between discrete nanocrystals are mainly van der Waals forces. Possible coordinative crosslinking and intergrowth between the nanocrystals may occur, though no conclusive evidence exists at present.^42^ In this case, transmission electron microscopy (TEM), scanning electron microscopy (SEM) and atomic pair distribution function (PDF) analysis indicated the absence of any other interactions, or indeed a separate amorphous or crystalline phase, which holds the crystalline nanoparticles together at the interface.

The MOF gel fabricated by Bennett *et al.* exhibits a viscoelastic property, and requires certain (external) stress to overcome internal interparticle interactions. Both flowing and non-flowing gels were fabricated, by controlling the crystalline volume fraction within the gel (Fig. 2). Interconversion between the two states was also achieved, for example by removing excess solvent *via* centrifugation and reducing the viscosity of a flowing gel sufficiently to form a non-flowing gel. Conversely, by applying enough shear forces, a non-flowing gel can be evenly redispersed in a larger solvent volume to form a flowing gel.^42^

Other researchers have converged upon the same mechanism for MOF gel formation.^12^,^41^ They also state that gelation is a consequence of the high initial nucleation rate of discrete MOF nanoparticles: the strong coordinative bonding between metal ions and organic ligands rapidly facilitates the assembly of MOF clusters, which then aggregate to form nanocrystals. Small crystalline MOF nanoparticles (<100 nm) have been experimentally observed at the early stages of gelation.^41^ Here, the dominance of supramolecular assembly over crystal growth leads to aggregation of particles and the formation of a gel. The key is to find the conditions under which the formation of nanocrystals can allow gelation to outcompete crystal growth and subsequent precipitation.

The formation process has been experimentally verified by monitoring the gel formation process of Al–BDC using solid-state NMR which showed identical subunit structure and metal node chemical environments during different stages of the gelation.^41^ Furthermore, *ex situ* PXRD of the gel contained Bragg peaks corresponding to the positions expected from MIL-53(Al). This also suggests the presence of crystalline components at the early stages of crystallization, and the broadness of the diffraction peaks suggests that crystalline particles have a size of around 10.4 nm based on Scherrer broadening analysis.^41^

PXRD can be useful in characterizing MOF gels, though data can often be misinterpreted and should be used in conjunction with other techniques. The scattering from crystalline nanoparticles can be very broad, though remains centred around the positions of the Bragg reflections. This must be differentiated from the broad diffuse scattering that is associated with amorphous materials. Sufficiently small nanoparticles can give rise to scattering that can easily be mistaken for an amorphous material. In these instances TEM, PDF analysis and extended X-ray absorption fine structure (EXAFS) analysis are useful.^44^

### Synthetic control of MOF gels

MOF gel formation consists of two separate steps: the nucleation of a large amount of discrete MOF nanoparticles, and subsequent gelation to form a colloidal network structure. Both aspects require careful regulation, especially the latter which usually competes against more thermodynamically favourable processes of precipitation. A key objective is thus to find a suitable set of conditions under which initial nucleation and subsequent gelation dominate. Bennett *et al.* highlighted several parameters necessary for the formation of a set of Zr-based MOF gels, including UiO-based series, MOF-801, MOF-808 and NU-1000.^42^ Here, the choice of metal source, the presence of water and concentration of the reactant were all found to play key roles.

In the case of using ZrOCl_2_·8H_2_O as a metal source, gels were favoured upon increasing reactant concentrations. Products progressed from microcrystalline powders at low concentration, to “flowing” gels and eventually to “non-flowing” opaque gels. Within a diluted precursor solution, the limited number of nuclei tend to grow and precipitate as large MOF particles rather than forming gels. The beneficial influence of high reactant concentration upon gel formation aligns with classical crystallization theory, where the nucleation rate is exponentially dependent on the reactant supersaturation.^45^ Conversely, lower initial concentrations of reactants in solution results in crystallization, linked to decreased nucleation relative to crystal growth.^42^

On the other hand, when ZrCl_4_ is used as the metal source, increasing reactant concentration alone is not sufficient to induce gelation, and water is required.^42^ This is not the case for the ZrOCl_2_·8H_2_O, where the water content of the precursor appears enough to promote initial [Zr_6_O_4_(OH)_4_]^12+^ cluster formation. Higher nucleation propensity and subsequent smaller MOF nanoparticles can therefore be expected with the progressive addition of water in the reactant solution.^46^,^47^

At the same time, controlling the gelation kinetics, rather than the initial nucleation, can be the dominating factor for some MOF gel systems. Li *et al.* prepared a series of Al(iii), Fe(iii), Ga(iii) and In(iii) MOF gels with carboxylic acid ligands, including H_3_BTC and H_2_BDC. While the metal–ligand precursor mixture remains stable at room temperature, gelation was observed to occur at an elevated temperature.^41^ This was rationalized by the higher temperatures facilitating greater reversibility of the coordination bonding, making it comparable to the weak non-covalent interactions which lead to gelation.

Extensive work has also been performed on the formation of HKUST-1 gels. For example, the deprotonation of the H_3_BTC ligand by KOH prior to addition of a Cu^2+^ solution results in a gel product, whilst the use of NaOH, LiOH or NEt_3_ does not.^48^ Solvent choice is also important, as gelation is observed with DMSO, though not DMF, ethanol or methanol.^49^ This was ascribed to stronger interactions between DMSO and Cu^2+^, which can facilitate the supramolecular association–dissociation process and results in the gelation of metal–organic clusters.^49^ The balance between gelation, and crystallization in MOFs is thus multi-faceted and influenced by multiple competing factors. At present, a detailed understanding is only gained on a case-by-case basis.

### Routes to MOF monoliths

The word monolith is derived from the ancient Greek word monolithos, meaning a single block of stone. Thus, a monolith of a material is of macroscopic size, with a continuous morphology.^50^ Conventionally, monolithic structures are categorized into organic polymeric, and inorganic-based categories.^51^,^52^ Early literature used a series of alternative names to describe these polymeric materials, such as “continuous polymer bed”,^53^ “continuous polymer rod”^54^ and “continuous column support”.^55^ A functionalized cellulose sponge was, however, termed ‘monolithic’ in 1993.^56^ The application of such sponges has mostly been as the stationary phases in chromatography columns, but they are hampered by their limited mechanical stability, which can compromise the porous structure over time under the high operating pressure of the chromatograph.^57^

Inorganic monoliths, for example the silica gel-based structures reported by Nakanishi and Soga in 1991,^58^ together with carbon, ceramic and metallic monoliths,^59^,^60^ have been the focus of much more extensive research. Their higher mechanical stabilities are advantageous in industrial processes, compared with beads or pellets, due to the reduced abrasion, lower flow resistance and pressure drop within the packed bed and flow through reactors.^52^ They are extensively used as catalyst supports for the combustion of methane, hydrogen and carbon monoxide, and in nitric monoxide reduction reactions.^61^–^63^

The assembly of hybrid materials, and specifically MOFs, into monolithic structures is advantageous in terms of (i) easier handling associated with higher structural rigidity, (ii) lower mass transfer resistance (associated with fewer surface barriers), and (iii) higher volumetric adsorption capacities and volumetric BET surface areas. The improved mechanical stability of continuous MOF bodies would also benefit devices in which the sorbent material faces friction against a container and/or operational vibrations.^40^ Such considerations have led to an increase in research on the fabrication and application of monolithic MOFs, with the goal of producing shaped microporous materials in the form of mechanically robust macrostructures, which retain their intrinsic microscale porous texture and inherent chemical and physical functionalities. The assembly, shaping and processing of such MOF monoliths is still however challenging, given that conventional solvothermal, microwave and mechanochemical synthetic methods result in fine powders.

### Non gel-derived methods of monolithic MOF formation

**Fig. 3:**
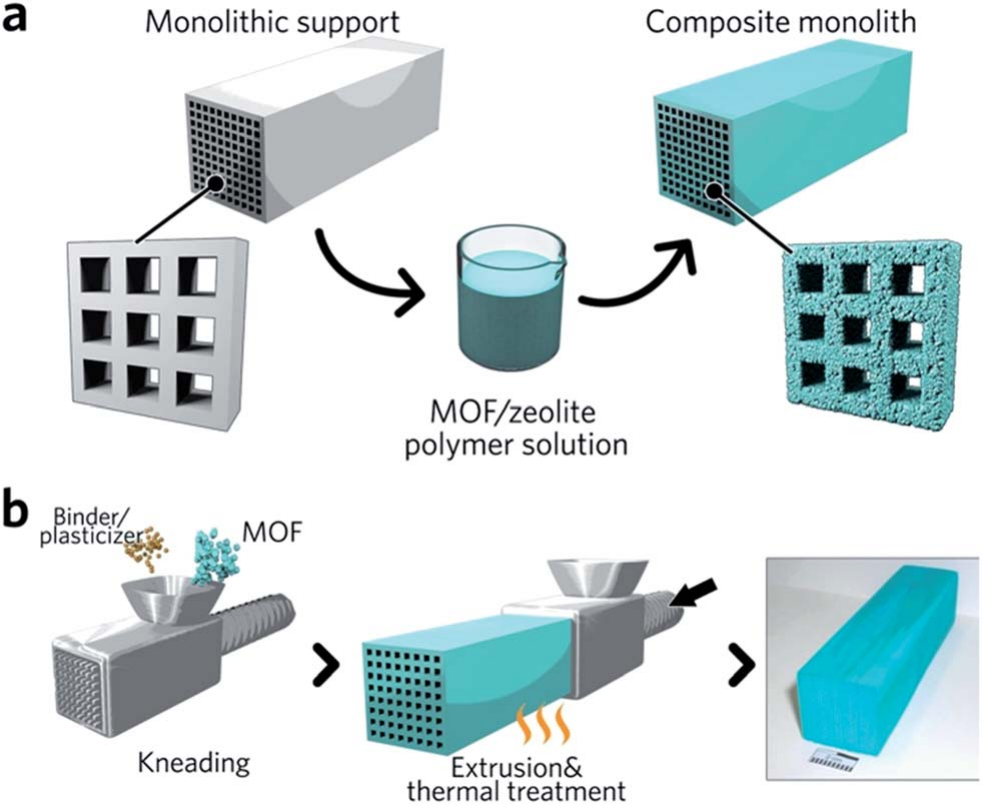
Fabrication of composite monolithic MOFs. (a) Wash coating to deposit MOFs on monolithic supports. (b) Extrusion approach for the fabrication of monolithic MOFs, with an optical photo of the resulting MOF monolith. Reproduced from ref. 67 with permission from the John Wiley & Sons, copyright 2010.

Monolithic MOF structures have been accessed *via* a variety of methods, including (i) the formation of composite materials through the use of binders, (ii) mechanical densification and (iii) coordination replication. Pioneering approaches for the formation of MOF composites include the coating of MOF-binder mixtures onto substrates, termed wash-coating (Fig. 3a), or growing MOFs on substrates in multiple cycle syntheses. Both inorganic scaffolds such as silica,^64^ and organic templates such as porous carbon^65^ have been used. PolyHIPE (high internal phase emulsion) foams are the most extensively investigated support to host MOF crystals, due to their commercial availability, good chemical stability and macroporous structures with high void fractions. For example, a multiple cycle impregnation procedure has been applied to fabricate an HKUST-1@HIPE composite monolith, which achieved a maximum MOF loading of 62.3 wt% after three impregnation cycles.^66^ Some composite monoliths can be fabricated using industrially available manufacturing processes. Küsgens *et al.*, for example, prepared monoliths of HKUST-1, by first homogenizing the MOF with binding and plasticization agents. This mixture was then extruded in an industrial ram extruder and cut to obtain 200 mm pieces (Fig. 3b).^67^

**Fig. 4:**
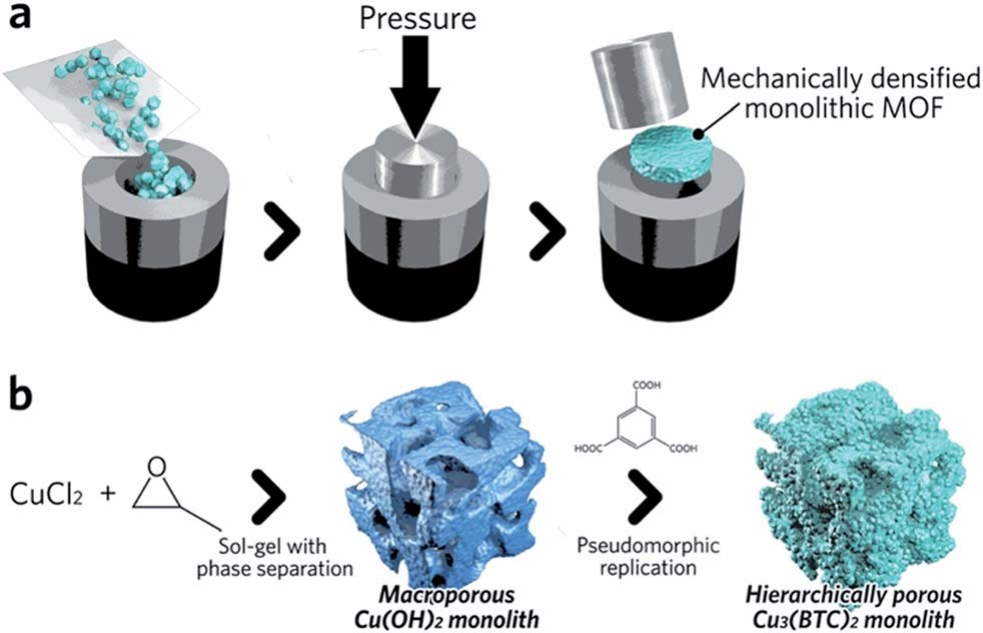
Schematic of methods available to fabricate single component MOF monoliths. (a) Mechanical densification and (b) coordination replication. (b) is reproduced from ref. 77 with permission from the Royal Society of Chemistry, copyright 2015.

Pathways to ‘pure’ MOF monoliths, *i.e.* those which contain only the active MOF component, are highly sought after. Mechanical densification is arguably the most straightforward technique amongst these. Typically, MOF powders are filled into a cylindrical die, which is closed using a dowel. The assembly is then placed between the jaws of an hydraulic press to apply external pressure (Fig. 4a).^68^ The compressed samples may have an improved volumetric gas adsorption capacity, together with an increased bulk density *versus* the free powder form (*e.g.* 0.09 and 0.23 cm^3^ (STP) cm^–3^ for powder and pellets, respectively, of UiO-66).^69^

A key objective of any effective compaction process is to reduce the intergranular void volume without destroying the intrinsic microporous structure. The optimum conditions required for compaction without structural collapse have been shown to be highly dependent upon the exact identity of the MOF. For example, UiO-66 has been considered as one of the most “rigid” MOFs due to its high degree of coordination between Zr–O secondary building units (SBUs) and organic ligands (each Zr_6_O_4_(OH)_4_ cluster is coordinated to 12 organic linkers).^70^ The minimal shear modulus of the framework is an order of magnitude higher than HKUST-1, where each copper atom in the paddlewheel SBU is coordinated by one water molecule and four oxygens from organic linkers.^71^,^72^ The UiO-66 structure remains unaffected upon pressurization to 700 MPa, whereas HKUST-1 experiences a loss of ∼50% in its BET surface area after densification at just 70 MPa.^73^,^74^ Slower pressurization rates and the presence of solvent within the MOF micropores have both been reported to be beneficial for the preservation of MOF microporosity under pressure.^69^ Recently, a more advanced pressure-assisted sintering technique (field-assisted sintering technique, FAST) has been introduced to fabricate monolithic ZIF-4 (Zn(Im)_2_, where Im = imidazolate) tablets with adjustable microporosity and macroporosity.^75^

A second approach to the fabrication of single component monoliths is based on the morphological replacement of a shaped, sacrificial, metal oxide mesoscopic template (Fig. 4b). This is also known as a pseudomorphic mineral replacement, in more general terms: the metastable metal oxide product dissolves at the solid/liquid interface, and immediately re-crystallizes as a more thermodynamically stable crystalline phase at the same site in the presence of organic ligands.^76^ For example, Cu(OH)_2_ monoliths have been used as a template for fabricating monolithic HKUST-1.^77^ In this case, full conversion of a Cu(OH)_2_ precursor under mild conditions within 6 min was observed.^77^

### Sol–gel MOF monoliths

Another important approach to the formation of pure monolithic MOF species is *via* the MOF gel state. Solvent removal from a MOF gel affords a monolithic MOF state, in which the absence of an inert material renders questions of active component loading obsolete, and avoids the issue of pore-blocking. They also provide advantages compared to monoliths prepared by densification, in that processing *via* the gel state allows greater control over product morphology, and consideration does not need to be paid to conditions which avoid structural collapse.^42^

The formation of sol–gel monoliths of MOFs is not new. For example, Kaskel *et al.* formed monolithic bodies of Fe-BTC in 2009,^78^ followed by the work of Furukawa and others on HKUST-1 and Al-multicarboxylate systems.^41^,^77^ Recently, Fairen-Jimenez, Bennett and others have reported sol–gel monoliths derived from Zr-based and HKUST-1 MOF gels.^12^,^42^,^79^ In the latter case, an increased capacity for volumetric adsorption of methane of 259 cm^3^ (STP) cm^–3^ was noted for the monolithic state over the crystalline powder material. The formation and structures of both MOF gels, and their resultant monolithic phases, are however relatively poorly understood, especially when compared with those prepared by conventional inorganic sol–gel processes, such as carbon, titania, silica and alumina.^80^

### Monolith formation from gels

In short, removal of solvent from a MOF gel improves the likelihood of nanoparticle aggregation, and the resultant formation of bulk monolithic structures. The non-uniform, non-covalent interactions between nanoparticles result in large, interparticulate void spaces, in addition to the nanopores within each particle. The presence of this hierarchical porosity facilitates rapid mass transfer, as well as high adsorptive capacities. As discussed, the formation of nanoparticles is an important pre-requisite for the formation of MOF gels and is, therefore, also an influential factor in formation of sol–gel MOF monoliths. For example, several HKUST-1 gels were created containing initial particle sizes of 51 to 73 nm. Gels composed of larger particles were found to form a monolith only after drying at low temperatures, whereas those gels formed of smaller initial particles were able to be heated at either low or higher temperatures to remove the solvent, and still formed monolithic pieces. During the drying process, there exists mechanical stress at the gas–liquid meniscus interface due to the surface tension. Therefore a slower solvent removal rate, or smaller primary nanoparticles can better accommodate this stress, leading to the formation of dense MOF monolith.^12^

**Fig. 5:**
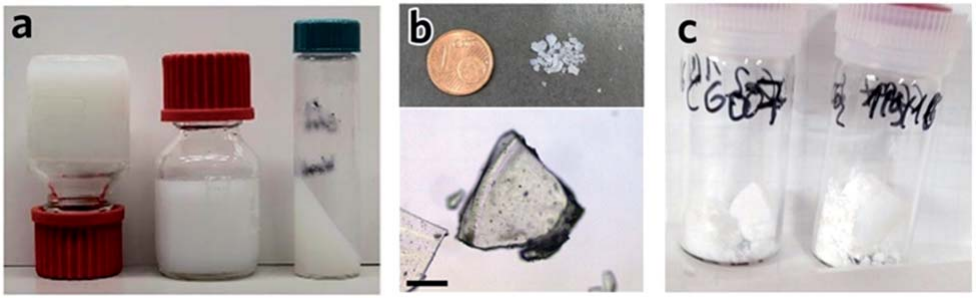
Transforming MOF gel to MOF monolith. (a–c) Optical photos of (a) “non-flowing” UiO-66 gel, (b) UiO-66 xerogel fabricated from UiO-66 gel by solvent removal at high temperature in air, and (c) UiO-66 aerogel fabricated with UiO-66 gel solvent removal by critical CO_2_ drying. Reproduced from ref. 42 with permission from the Royal Society of Chemistry, copyright 2017.

In other systems, mesopore size, and resultant monolith morphology, may be altered through alternative solvent removal conditions. For example, solvent evaporation from a UiO-66 gel (Fig. 5a) under high temperatures in air is likely to result in the formed monolith (xerogel) experiencing a significant shrinkage in volume due to the capillary force exerted during the drying process (Fig. 5b), which reduces mesopore size.^78^ In comparison, slow and controllable removal of the solvent from the gel produces aerogels (Fig. 5c), *e.g.* by sub/supercritical CO_2_ extraction,^41^,^42^ results in avoidance of mesopore collapse and formation of high porosity, low density and large internal surface areas. Different macroscopic structures have also been prepared *via* different approaches of solvent removal for HKUST-1 monoliths.^12^ Materials of intermediate porosity have been formed from conventional inorganic sol–gels,^81^–^84^ through freeze-drying. Such cryogels have not yet however been formed from MOF gels.

## Monolith structure and properties

**Fig. 6:**
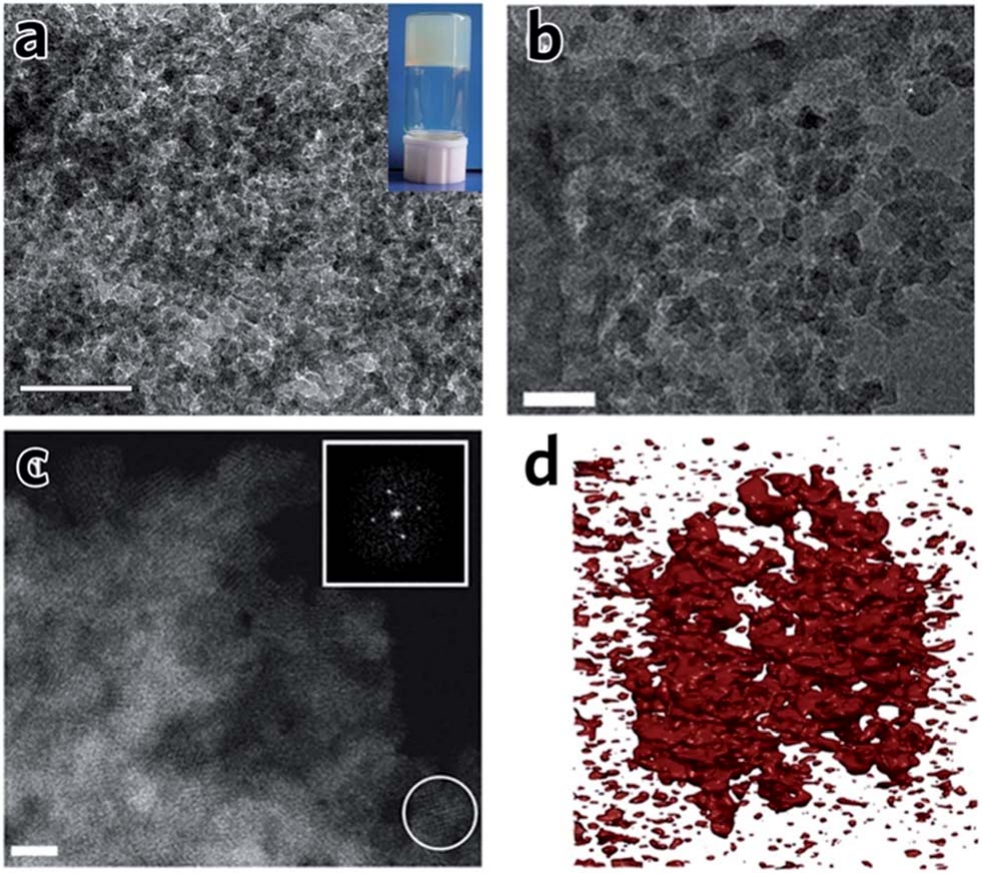
TEM images and tomography of MOF monoliths. (a) TEM image of an Al–BDC aerogel. The inset is the photo of the MOF gel before solvent extraction. The scale bar is 100 nm. (a) is reproduced from ref. 41 with permission from the Springer Nature, copyright 2013. (b) TEM image and (c) ADF-STEM image of UiO-66 xerogel, showing the randomly packed nanocrystals with interparticle pores. The scale bar is (b) 50 nm and (c) 10 nm. (d) Electron tomographic reconstruction of the UiO-66 xerogel particle of around 100 nm in size. Solid matter is represented in red. (b)–(d) are reproduced from ref. 42 with permission from the Royal Society of Chemistry, copyright 2017.

TEM experiments on monoliths of Al–BDC 41 and UiO-66 42 clearly indicate that the structures are comprised of irregularly packed nanoparticles (Fig. 6a and b), *i.e.* there is no change to the structure of the nanoparticles upon drying of the precursor MOF gel. In particular, for a UiO-66 xerogel, annular dark field (ADF) scanning transmission electron microscopy (STEM) shows the monolith consists entirely of aggregated crystalline nanoparticles of around 10 nm in size (Fig. 6c).^42^ The presence of mesoporous interparticle spaces (10–30 nm) is confirmed by tomographic reconstruction from electron microscopy data (Fig. 6d).

**Fig. 7:**
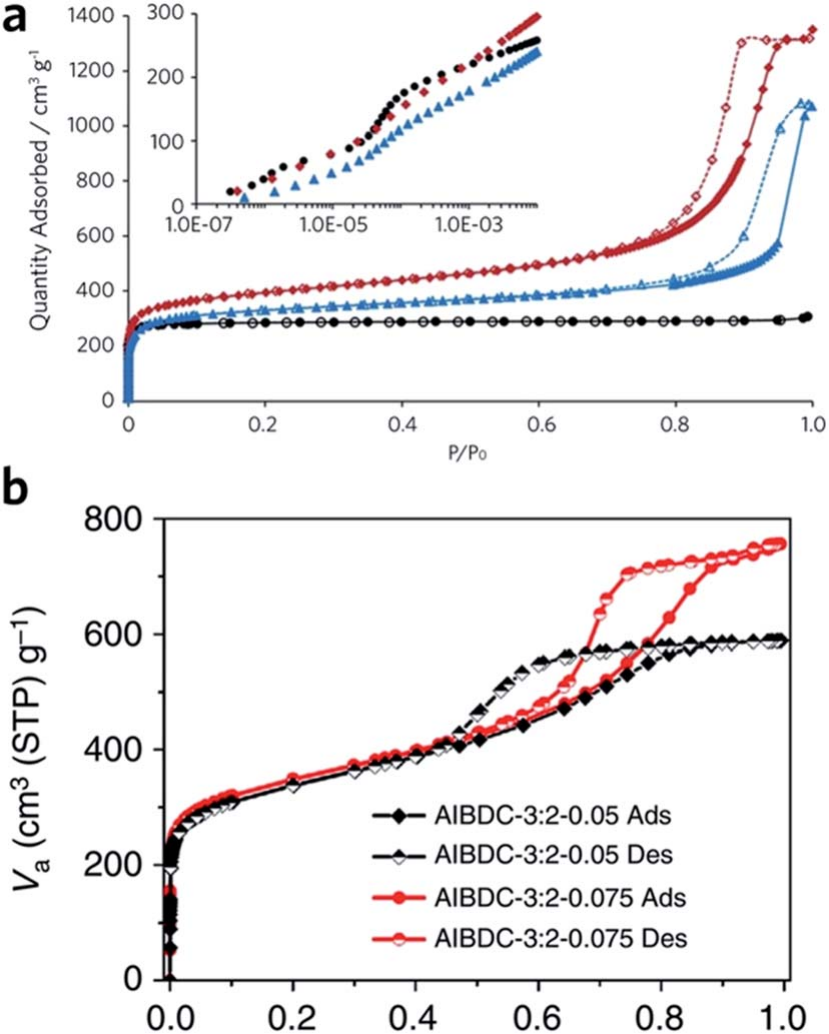
Nitrogen physisorption isotherms at 77 K for UiO-66 crystalline powder (back), xerogel (red) and aerogel (blue). Full symbols are adsorption branch and open symbols are desorption branch. The inset is a logarithmic representation of the adsorption branch at low pressure region, showing the step-wise nitrogen uptake by smaller tetrahedral (6 Å) and larger octahedral cages (8 Å). (a) is reproduced from ref. 42 with permission from the Royal Society of Chemistry, copyright 2017. (b) Nitrogen physisorption isotherms for Al–BDC monoliths fabricated with different precursor concentrations. (b) is reproduced from ref. 41 with permission from the Springer Nature, copyright 2013.

Sol–gel monolithic MOFs typically exhibit Type IV, mesoporous, N_2_ sorption behaviour, showing pore condensation with adsorption–desorption hysteresis at high pressures (Fig. 7a and b). This observation confirms the presence of both micro- and mesoporosity.^41^,^42^ Based on N_2_ isotherm and mercury intrusion measurements, monolithic UiO-66 possesses 3–4 times higher total porosity compared with the bulk MOF powder, which is mainly ascribed to the presence of considerable mesoporosity.^42^ Monoliths formed from smaller nanoparticles may, however, exhibit smaller surface areas, due to a reduction in micropore surface area and volume compared to the external surface area.^85^

It has also been proposed in the case of HKUST-1 that, unlike other MOF monoliths, a dense structure is formed upon solvent evacuation. A mechanism was proposed whereby the gradual removal of solvents under mild temperatures allows the unreacted precursors to nucleate at the interstitial space interface, leading to epitaxial growth to fill the space, forming a near-continuous structure. Within this monolithic sample, there exists no boundary or interphase between the primary particles, resulting in a continuous phase without mesopores.^12^ Unexpectedly, the densification does not significantly compromise adsorption kinetics. For example, both powdered and monolithic samples show rapid equilibrium with methane, with only slightly slower transport diffusivity for the dense MOF due to the absence of mesoporosity. The monolithic MOF exhibits improved mechanical stability towards irreversible plastic deformation (as suggested by the significantly higher Young's Modulus of the material), which can be attributed to its higher density.^12^

As in microcrystalline MOF powders, the preservation of microporosity is closely related to the nature of the organic ligand. For example, for a monolith fabricated with Cr^3+^ and different bridging carboxylic acids, the incorporation of H_2_BDC or H_3_BTC leads to BET surface area of over 400 m^2^ g^–1^. However, the monoliths fabricated with bulkier 9-fluorenone-2,7-dicarboxylic acid (H_2_FDC) and bent ligand 9,10- anthracenedicarboxylic acid (H_2_ADC) have significantly lower BET surface areas. A more significant loss of the surface area is observed with ligands possessing an aliphatic substituent, indicating the microporosity may also be reduced by pore blockage from aliphatic side groups.^86^

### Architectural and macroscale shaping of MOF monoliths

The similarity between MOF and inorganic nanoparticle sol–gel processes has inspired the use of supramolecular templating during the gelation stage, in order to regulate mesostructure.^87^ The surfactant cetyltrimethylammonium bromide (CTAB), together with auxiliary agent 1,3,5-trimethylbenzene (TMB), has been applied in this way and resulted in monoliths possessing a narrower mesopore distribution.^41^ Other soft (amphiphiles, block copolymers, ionic liquids, biopolymers and biomacromolecules) and hard (colloidal nanoparticles, bacterial filaments or cellulose nanocrystals) templates may also be able to regulate MOF monolith mesostructures,^80^ though reports in this area are still rare.

**Fig. 8:**
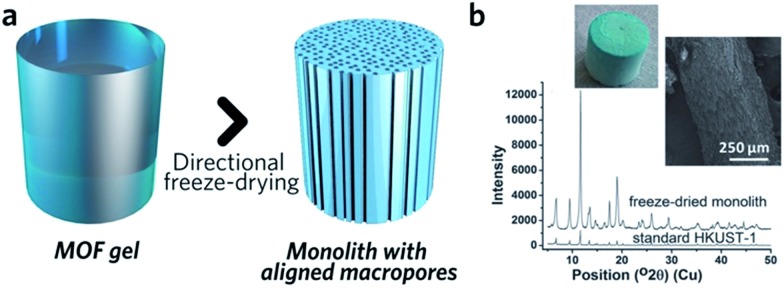
Freeze drying to introduce aligned macropores to monolithic MOF. (a) Schematic diagram of the freeze-drying process. (b) PXRD, optical photo and SEM image of the HKUST-1 monolith with aligned macropores. Reproduced from ref. 89 with permission from the Royal Society of Chemistry, copyright 2015.

Control over the macroporous structure of MOF monoliths is harder to achieve. One promising avenue may be to employ a controlled freeze, and subsequent freeze-drying, approach. This technique, also known as ice-templating, has been applied for the fabrication of various porous materials.^88^ Work on HKUST-1 demonstrated that it is possible to fabricate a monolithic species with aligned pores, *via* a directional freezing process (Fig. 8). The aligned macropores offer enhanced mass transport and low-pressure drop across the monolithic column. Water and organic solvents (*e.g.* DMSO) may also be applied as frozen templates, and further control of macropore size exhibited by varying freezing rate or temperature. Formation of the inter-connected monolith structure in this case was, however, reliant upon the bridging of separated nanocrystals by the remaining reactants, as, unlike slow evaporation, freeze drying of the gel alone did not form a continuous network.^89^

The gel state does however provide a flexible setting for the fabrication of solid monolithic bodies with pre-defined shapes.^25^ MOF gels usually exhibits a viscoelastic behaviour, and can be shaped using a specific mould, forming monolithic solids for different industrial settings like heterogeneous catalysis, adsorption and membrane/film fabrication.^90^ Some gels exhibit stimuli-responsive behaviour: reversible transitions between gel and colloidal suspension (slurry-like fluid) have been observed, which can be triggered by thermal treatment or mechanical shaking (thixotropic responsiveness).^90^ In addition, the microscale morphological structures of MOF gels can be shifted by adding metal ion or organic ligand into the formed MOF gel, *e.g.* transforming HKUST-1 from a granular to fibrous shape.^48^

Bennett *et al.* attempted to produce shaped monolithic species of UiO-66 in the form of spherical, monodispersed beads. An oil-drop granulation process was employed, where the flowing form of a MOF gel in DMF solvent was dispensed by a perfusion pump into immiscible hot silicone oil.^42^ The droplets underwent syneresis, and formed a xerogel after 10 min at 150 °C. Subsequent annealing in air leads to the formation of the uniform monolithic MOF beads. The final monolith preserved the original MOF crystalline structure and porosity, and sizes were regulated by using different sized needles to dispense the gel solution. The significant drawback of the method was however the time taken to produce the beads.

## Applications

### Guest adsorption

The critical limitation of using natural, or hydrogen gas as a transportation fuel is the low storage density at ambient temperature, which leads to a significantly lower volumetric energy density compared with traditional fossil fuels.^91^ The US DOE has set an ambitious volumetric storage target for methane of 263 cm^3^ (STP) cm^–3^ at room temperature and 65 bar.^92^ In terms of the methane and hydrogen storage, some MOFs with large surface areas also exhibit good promise for practical application. For example, DUT-49 (Cu_2_(BBCDC), where BBCDC = 9,9′-([1,1′-biphenyl]-4,4′-diyl)bis(9*H*-carbazole-3,6-dicarboxylate)), with a specific surface area of 5476 m^2^ g^–1^, showed an H_2_ excess uptake of 80 mg g^–1^ at 77 K under 50 bar, and an exceptionally high methane storage capacity of 308 mg g^–1^ at 298 K under 110 bar. In reality however, the problem of low packing density and low mechanical strength may limit the applicability of MOFs.^93^

**Fig. 9:**
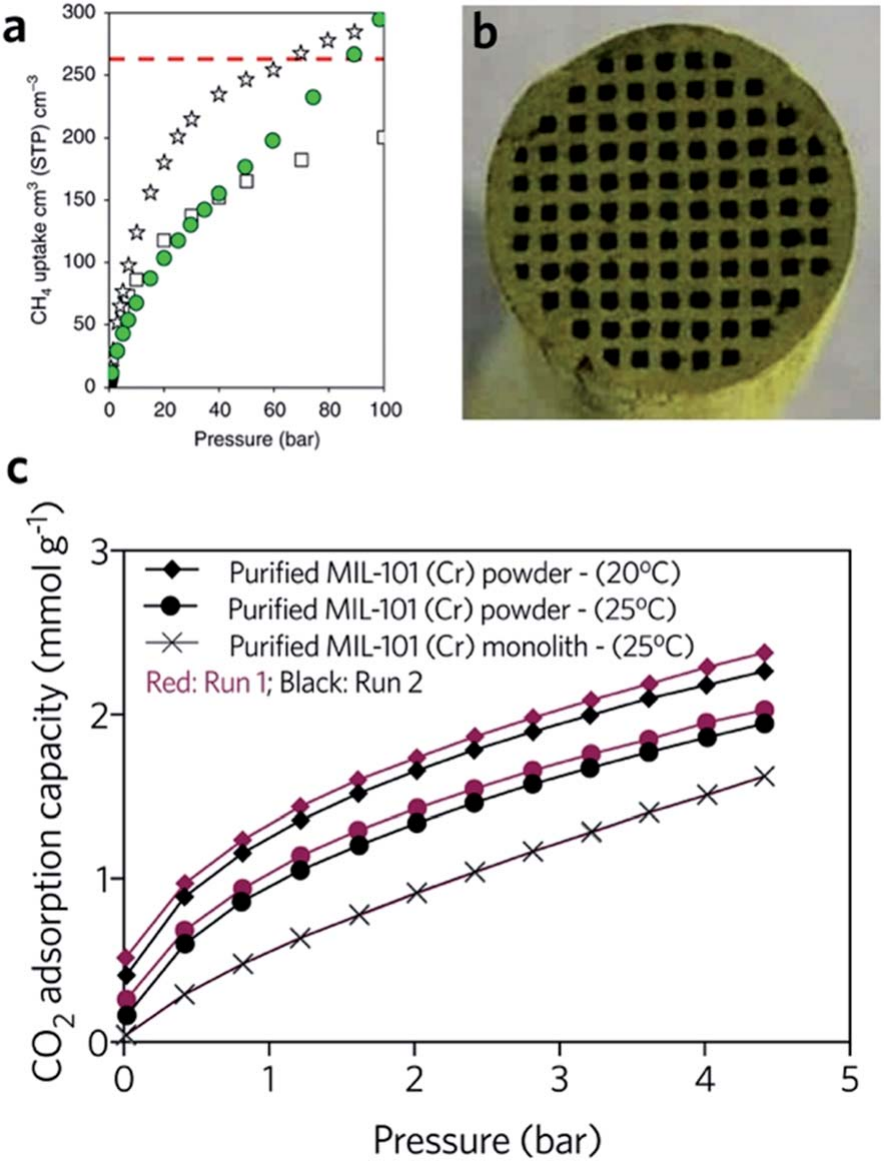
Monolithic MOF for gas storage and gas adsorption. (a) Methane gas adsorption at 298 K for HKUST-1 (white stars) and UiO-66 (green circles) monolithic materials. Comparison is made against computationally simulated purely microporous/defect-free UiO-66 (white squares) at 298 K. The DOE target of methane uptake is represented by the red dashed line in the graph. Reproduced from ref. 79 with permission from the Springer Nature, copyright 2019. (b and c) CO_2_ adsorption for biogas upgrade with MIL-101 (Cr) monolith. (b) Optical image of the cross-sectional image of the MIL-101 (Cr) monolith, and (c) CO_2_ adsorption isotherms of MOF powders and MOF monolith. (b and c) are reproduced from ref. 94 with permission from the Elsevier, copyright 2015.

The hierarchical porous structure of sol–gel MOF monoliths is crucial for the reversible adsorption of small guest molecules, with minimal mass transport resistance. The presence of a mesoporous structure also facilitates a higher gas adsorption capacity, especially for condensable gases.^41^ Fairen-Jimenenz *et al.* reported a key example, where a pure monolithic sample of HKUST-1 was demonstrated to meet the DOE target for methane uptake (Fig. 9a).^79^

In another example, a monolithic MIL-101 (Cr) (Cr_3_O(OH)(BDC)_3_(H_2_O)_2_) sample was reported by Hong *et al.*, exhibiting an equilibrium CO_2_ adsorption capacity of 1.95 mmol g^–1^ under ambient conditions. The low-pressure drop effect for CO_2_ when packed into an adsorption column, combined with stability under humid feed gas conditions, demonstrated promise for removal of CO_2_ from industrial gas streams. In comparison, the loss of CO_2_ uptake capacity for a column filled with pure MIL-101 (Cr) is observed during cycling (Fig. 9b and c). One drawback of the use of the monolith is that, with increasing effective loadings (from 60 to 75 wt%), longer times are required to reach equilibrium in the dynamic adsorption process, indicating an increase in resistance to mass transfers.^94^ A fine balance between adsorptive kinetics and capacity is therefore required.

Wastewater treatment using monolithic MOFs mainly focuses on micropollutants (*e.g.* pharmaceutically active compounds, pesticides, endocrine disrupting agents), which are difficult to remove using conventional water treatment techniques such as filtration, sedimentation, membrane bioreactors and even advanced oxidization.^95^,^96^ It is proposed that monolithic MOFs can be dispersed in wastewater for the adsorption of such pollutants, and then easily recovered *via* mesh filtration due to their mascroscopic size. Then the active monolithic MOF can be regenerated after micropollutant removal, usually by solvent extraction.^97^ For example, microcystine is produced by cyanobacteria in water, and can cause liver damage even at low dosage. It is a chemically stable compound that displays resistance to conventional water treatment procedures once produced. Monolithic MIL-100 (Al) (Al_3_O(OH)(BTC)_2_(H_2_O)_2_), in the form of both aerogels and xerogels, has been applied for the adsorption of microcystine from aqueous environments, and was reported to possess a larger adsorption capacity and faster adsorption rate than other comparable porous materials including mesoporous silica SBA-15 and active carbon.^97^

### Separations

HKUST-1 monoliths have, in the past, been employed as the stationary phase within a chromatography column, and used in liquid phase separations.^98^ For example, fast separation of ethylbenzene and styrene by an HKUST-1 monolith is achieved within 2 min, with a back pressure of 134 bar. Notably, the monolith significantly outperforms powdered MOF counterparts, where over 2.5 h is required to achieve the separation.^98^ This rapid separation originates mainly from specific interactions between the analyte and the stationary phase, combined with the dynamic flow of the mobile phase through the monolithic MOF porous structures. The column also exhibits satisfactory performance over a long-term operation (6 weeks with over 60 injections) and under a high back pressure of 300 bar.^99^

**Fig. 10:**
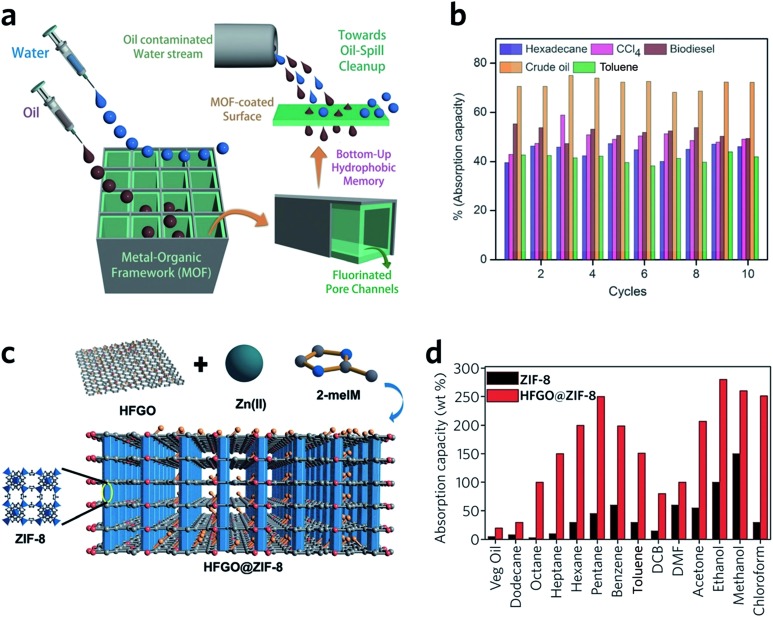
Oil–water separation using monolithic MOF materials. (a) Schematic diagram of oil-water separation with the MOF containing fluorinated linkers. (b) Oil absorption capacities of the monolithic MOF containing fluorinated linkers. (a and b) are reproduced from ref. 101 with permission from the John Wiley & Son, copyright 2016. (c) Illustration showing the concept of the highly fluorinated graphene oxide and ZIF-8 composite. (d) Absorption of oil and organic solvents with ZIF-8 and composite ZIF-8. (c and d) are reproduced from ref. 102 with permission from the John Wiley & Sons, copyright 2016.

Monolithic MOFs with large surface areas, tuneable wettabilities and good water resistance have been shown to play an important role in oil–water separations.^100^ In a recent study, a superhydrophobic (water contact angle of above 170°), water-stable MOF containing Cu^2+^ and hexafluorinated dicarboxylate linkers was prepared. After coating on a porous support, the monolithic MOF film exhibits excellent oil absorption capacities and reusability, during the separation of hexadecane, bio-diesel, toluene and crude oil from water (Fig. 10a and b).^101^ Composites may also be applied to tackle the same problem. For example, highly fluorinated graphene oxide (HFGO) has been mixed into a ZIF-8 precursor solution, where the GO allows selective nucleation of ZIF-8 on its surface, and serves as structure directing agent in the production of a mesoporous structure. The resultant material exhibits high absorptive selectivity and rapid kinetics for oil absorption from water. The composite has an excellent oil absorption capacity of 150 to 500% of its initial dry weight, which surpasses pristine MOF gels and all other reported crystalline pure MOF materials (Fig. 10c and d).^102^

### Further applications

Monoliths have also been suggested to possess promise in other areas. For example, post-synthetic modification has been used to produce luminescent sensing xerogels.^103^ In the area of catalysis, on top of the controllable hierarchical structure mentioned above, monolithic MOFs have several inherent advantages. Due to the nature of the MOF gel formation process, the formed primary nanocrystals may contain a large proportion of under-coordinated metal sites, which are expected to promote their catalytic and adsorptive efficacy.^90^,^104^ For example, the presence of coordinative unsaturated Cu^2+^ sites endows HKUST-1 with attractive Lewis acid catalytic capabilities, such as Diels–Alder reactions, cyclization of citronellal and the Friedländer reaction.^9^,^105^–^107^ The high surface area of MOFs can also locally concentrate the reactants to promote the reaction. Therefore, there are driving forces to assemble MOFs into practical catalytic reactors. The continuous flow catalytic reactors enabled by monolithic MOFs offer easy implementation, direct product separation, long-term stable performance and easier catalyst regeneration. In addition, due to the good preservation of the nanosized MOF crystals, the catalytic efficiency can be usually higher than the conventional batch reactors with a slurry of MOF particles.^64^

In addition, the functional groups or the micropores within the MOFs can serve as anchoring sites for catalytically active guest species.^108^ Mehta *et al.* reported the fabrication of a chemically and mechanically robust monolithic ZIF-8 material *via* a sol–gel process. Photocatalytically active SnO_2_ nanoparticles were encapsulated *in situ* within the monolith during the crystallization process. Compared with the pure MOF powders, the composites with monolithic MOFs demonstrated improved photocatalytic activities and reusabilities in dye degradation: the micropores of ZIF-8 provide size-exclusion for the organics, only allowing the diffusion of water and hydrogen peroxide as carriers for the indirect photo-degradation reaction. The structure and catalytic mechanism prevents the poisoning and leaching of the expensive catalytic nanoparticles.^109^

The application of MOF monoliths and gels in energy-related fields may be an intriguing avenue for future research. For example, redox-active Fe-based heteroatom electrodes have been prepared from Fe-based aerogels. By applying suitable carbonization temperatures, Fe_3_O_4_ active sites were maintained, and the final resultant electrodes exhibit stable performance over 5000 charge–discharge cycles with a high current density (8 A g^–1^).^110^

## Outlook

The fabrication, optimization and design of MOF monoliths is an intriguing field. It has shown great promise for improving the industrial viability of MOFs. However, there are significant areas for further work, and large knowledge gaps to bridge in order to fully exploit their potential. Our understanding of the formation mechanisms of pure MOF monoliths *via* sol–gel syntheses remains poor, and is limited to a case-by-case basis. In particular, the crystallization process and precise role of interactions between nanoparticles needs further exploration. A better understanding of these processes may enable the fabrication of pure sol–gel monoliths from a whole array of MOF structures.

It is important that possible misconceptions in the analysis of PXRD data are addressed, if the fields of MOF gels and amorphous MOFs are to be correctly delineated. In particular, we highlight that the absence of sharp diffraction peaks in powder diffraction data does not distinguish between extremely small nanoparticle-based structures, and those which are three dimensional amorphous solids. Electron microscopy, amongst other techniques, should be used in such cases to distinguish between the two and confirm, or rule out, the presence of crystalline MOF nanoparticles.^111^

The gel and monolithic states hold great promise for the future of materials discovery in the field. For example, Maspoch *et al.* recently demonstrated how several functional forms of ZIF-8 monoliths may be prepared using only small changes in reaction conditions,^112^ whilst Furukawa *et al.* have investigated the linking and self assembly of metal–organic polyhedra together, to form amorphous gel structures.^113^ Such reports highlight the potential of the MOF–gel state in the production of new functional materials.

The multi-stage hierarchical structure properties of MOF gels and monoliths may also facilitate tandem multi-stage catalytic reactions. However, bringing monolithic MOFs and gels closer to commercialization requires scalable fabrication. More effort, therefore, must be dedicated to investigating the fabrication techniques for mass production of the complicated monolithic structures, ideally with a solvent-free approach or a benign solvent synthesis process. High mechanical robustness of these materials are also needed. Further investigation is therefore required to fabricate monolithic MOFs which are able to withstand the application of external stress, without breaking up into their constituent nanoparticles.

It is abundantly clear from the examples presented in this perspective that there is still much to be discovered in the field of MOF gels and monoliths. However, with a greater understanding of MOF crystallization and gelation we shall be able to access a host of morphologically diverse monolithic materials, with highly tuneable hierarchical porosities, that are well suited to range of industrial applications.

## Conflicts of interest

There are no conflicts to declare.

## References

[cit1] Wang H., Zhu Q.-L., Zou R., Xu Q. (2017). Chem.

[cit2] Kim H., Yang S., Rao S. R., Narayanan S., Kapustin E. A., Furukawa H., Umans A. S., Yaghi O. M., Wang E. N. (2017). Science.

[cit3] DeCoste J. B., Peterson G. W. (2014). Chem. Rev..

[cit4] Kumar P., Kim K.-H., Kwon E. E., Szulejko J. E. (2016). J. Mater. Chem. A.

[cit5] Li X., Liu Y., Wang J., Gascon J., Li J., Van der Bruggen B. (2017). Chem. Soc. Rev..

[cit6] Kang Z., Fan L., Sun D. (2017). J. Mater. Chem. A.

[cit7] Li J.-R., Kuppler R. J., Zhou H.-C. (2009). Chem. Soc. Rev..

[cit8] Li H., Wang K., Sun Y., Lollar C. T., Li J., Zhou H.-C. (2018). Mater. Today.

[cit9] Gascon J., Corma A., Kapteijn F., Llabrés i Xamena F. X. (2014). ACS Catal..

[cit10] Ren J., Langmi H. W., North B. C., Mathe M. (2015). Int. J. Energy Res..

[cit11] Stock N., Biswas S. (2012). Chem. Rev..

[cit12] Tian T., Zeng Z., Vulpe D., Casco M. E., Divitini G., Midgley P. A., Silvestre-Albero J., Tan J.-C., Moghadam P. Z., Fairen-Jimenez D. (2017). Nat. Mater..

[cit13] Lee J., Farha O. K., Roberts J., Scheidt K. A., Nguyen S. T., Hupp J. T. (2009). Chem. Soc. Rev..

[cit14] García-García P., Müller M., Corma A. (2014). Chem. Sci..

[cit15] Morris R. E., Wheatley P. S. (2008). Angew. Chem., Int. Ed..

[cit16] Farha O. K., Özgür Yazaydın A., Eryazici I., Malliakas C. D., Hauser B. G., Kanatzidis M. G., Nguyen S. T., Snurr R. Q., Hupp J. T. (2010). Nat. Chem..

[cit17] Niaz S., Manzoor T., Pandith A. H. (2015). Renewable Sustainable Energy Rev..

[cit18] Rosi N. L. (2003). Science.

[cit19] Rowsell J. L. C., Yaghi O. M. (2005). Angew. Chem., Int. Ed..

[cit20] Valizadeh B., Nguyen T. N., Stylianou K. C. (2018). Polyhedron.

[cit21] Rubio-Martinez M., Avci-Camur C., Thornton A. W., Imaz I., Maspoch D., Hill M. R. (2017). Chem. Soc. Rev..

[cit22] Denny M. S., Moreton J. C., Benz L., Cohen S. M. (2016). Nat. Rev. Mater..

[cit23] Seoane B., Coronas J., Gascon I., Benavides M. E., Karvan O., Caro J., Kapteijn F., Gascon J. (2015). Chem. Soc. Rev..

[cit24] Zhu H., Yang X., Cranston E. D., Zhu S. (2016). Adv. Mater..

[cit25] Lim G. J. H., Wu Y., Shah B. B., Koh J. J., Liu C. K., Zhao D., Cheetham A. K., Wang J., Ding J. (2019). ACS Mater. Lett..

[cit26] Vilela S. M. F., Salcedo-Abraira P., Micheron L., Solla E. L., Yot P. G., Horcajada P. (2018). Chem. Commun..

[cit27] IUPAC Compendium of Chemical Terminology: Gold Book, ed. M. Nič, J. Jirát, B. Košata, A. Jenkins and A. McNaught, IUPAC, Research Triagle Park, NC, 2.1.0. edn, 2009.

[cit28] Zhang J., Hu Y. and Li Y., in Gel Chemistry, Springer Singapore, Singapore, 2018, vol. 96, pp. 61–118.

[cit29] Sumida K., Liang K., Reboul J., Ibarra I. A., Furukawa S., Falcaro P. (2017). Chem. Mater..

[cit30] Moghadam P. Z., Li A., Wiggin S. B., Tao A., Maloney A. G. P., Wood P. A., Ward S. C., Fairen-Jimenez D. (2017). Chem. Mater..

[cit31] Avrami M. (1939). J. Chem. Phys..

[cit32] Morris R. E. (2009). ChemPhysChem.

[cit33] Gualtieri A. F. (2001). Phys. Chem. Miner..

[cit34] Avrami M. (1940). J. Chem. Phys..

[cit35] Avrami M. (1941). J. Chem. Phys..

[cit36] Hancock J. D., Sharp J. H. (1972). J. Am. Ceram. Soc..

[cit37] Pradell T., Crespo D., Clavaguera N., Clavaguera-Mora M. T. (1998). J. Phys.: Condens. Matter.

[cit38] LaMer V. K., Dinegar R. H. (1950). J. Am. Chem. Soc..

[cit39] Yeung H. H.-M., Sapnik A. F., Massingberd-Mundy F., Gaultois M. W., Wu Y., Fraser D. X., Henke S., Pallach R., Heidenreich N., Magdysyuk O., Vo N. T., Goodwin A. L. (2018). Angew. Chem., Int. Ed..

[cit40] Tian T., Velazquez-Garcia J., Bennett T. D., Fairen-Jimenez D. (2015). J. Mater. Chem. A.

[cit41] Li L., Xiang S., Cao S., Zhang J., Ouyang G., Chen L., Su C.-Y. (2013). Nat. Commun..

[cit42] Bueken B., Van Velthoven N., Willhammar T., Stassin T., Stassen I., Keen D. A., Baron G. V., Denayer J. F. M., Ameloot R., Bals S., De Vos D., Bennett T. D. (2017). Chem. Sci..

[cit43] Draper E. R., Adams D. J. (2017). Chem.

[cit44] Billinge S. J. L., Levin I. (2007). Science.

[cit45] Girshick S. L., Chiu C. (1990). J. Chem. Phys..

[cit46] Ragon F., Horcajada P., Chevreau H., Hwang Y. K., Lee U.-H., Miller S. R., Devic T., Chang J.-S., Serre C. (2014). Inorg. Chem..

[cit47] Schaate A., Roy P., Godt A., Lippke J., Waltz F., Wiebcke M., Behrens P. (2011). Chem. – Eur. J..

[cit48] Liao P., Fang H., Zhang J., Hu Y., Chen L., Su C.-Y. (2017). Eur. J. Inorg. Chem..

[cit49] Chaudhari A. K., Han I., Tan J.-C. (2015). Adv. Mater..

[cit50] Gvishi R., in The Sol-Gel Handbook, ed. D. Levy and M. Zayat, Wiley-VCH Verlag GmbH & Co. KGaA, Weinheim, Germany, 2015, pp. 317–344.

[cit51] Schoenherr R. M., Ye M., Vannatta M., Dovichi N. J. (2007). Anal. Chem..

[cit52] Williams J. L. (2001). Catal. Today.

[cit53] Hjertén S., Yi-Ming Li Y., Liao J.-L., Mohammad J., Nakazato K., Pettersson G. (1992). Nature.

[cit54] Svec F., Frechet J. M. J. (1992). Anal. Chem..

[cit55] Fields S. M. (1996). Anal. Chem..

[cit56] Kennedy J. and Williams O., Cellulosics: Materials for Selective Separations and other Technologies, Ellis Horwood, 1993.

[cit57] Zou H., Huang X., Ye M., Luo Q. (2002). J. Chromatogr. A.

[cit58] Nakanishi K., Soga N. (1991). J. Am. Ceram. Soc..

[cit59] Walsh Z., Paull B., Macka M. (2012). Anal. Chim. Acta.

[cit60] Pfaunmiller E. L., Paulemond M. L., Dupper C. M., Hage D. S. (2013). Anal. Bioanal. Chem..

[cit61] Rezaei F., Webley P. (2010). Sep. Purif. Technol..

[cit62] Siemund S., Leclerc J. P., Schweich D., Prigent M., Castagna F. (1996). Chem. Eng. Sci..

[cit63] Govender S., Friedrich H. (2017). Catalysts.

[cit64] Sachse A., Ameloot R., Coq B., Fajula F., Coasne B., De Vos D., Galarneau A. (2012). Chem. Commun..

[cit65] Qian D., Lei C., Hao G.-P., Li W.-C., Lu A.-H. (2012). ACS Appl. Mater. Interfaces.

[cit66] Schwab M. G., Senkovska I., Rose M., Koch M., Pahnke J., Jonschker G., Kaskel S. (2008). Adv. Eng. Mater..

[cit67] Küsgens P., Zgaverdea A., Fritz H.-G., Siegle S., Kaskel S. (2010). J. Am. Ceram. Soc..

[cit68] Oh H., Lupu D., Blanita G., Hirscher M. (2014). RSC Adv..

[cit69] Dhainaut J., Avci-Camur C., Troyano J., Legrand A., Canivet J., Imaz I., Maspoch D., Reinsch H., Farrusseng D. (2017). CrystEngComm.

[cit70] Su Z., Miao Y.-R., Zhang G., Miller J. T., Suslick K. S. (2017). Chem. Sci..

[cit71] Wu H., Yildirim T., Zhou W. (2013). J. Phys. Chem. Lett..

[cit72] Prestipino C., Regli L., Vitillo J. G., Bonino F., Damin A., Lamberti C., Zecchina A., Solari P. L., Kongshaug K. O., Bordiga S. (2006). Chem. Mater..

[cit73] Peterson G. W., DeCoste J. B., Glover T. G., Huang Y., Jasuja H., Walton K. S. (2013). Microporous Mesoporous Mater..

[cit74] Bambalaza S. E., Langmi H. W., Mokaya R., Musyoka N. M., Ren J., Khotseng L. E. (2018). J. Mater. Chem. A.

[cit75] Widmer R. N., Lampronti G. I., Kunz B., Battaglia C., Shepherd J. H., Redfern S. A. T., Bennett T. D. (2018). ACS Appl. Nano Mater..

[cit76] Reboul J., Furukawa S., Horike N., Tsotsalas M., Hirai K., Uehara H., Kondo M., Louvain N., Sakata O., Kitagawa S. (2012). Nat. Mater..

[cit77] Moitra N., Fukumoto S., Reboul J., Sumida K., Zhu Y., Nakanishi K., Furukawa S., Kitagawa S., Kanamori K. (2015). Chem. Commun..

[cit78] Lohe M. R., Rose M., Kaskel S. (2009). Chem. Commun..

[cit79] Connolly B. M., Aragones-Anglada M., Gandara-Loe J., Danaf N. A., Lamb D. C., Mehta J. P., Vulpe D., Wuttke S., Silvestre-Albero J., Moghadam P. Z., Wheatley A. E. H., Fairen-Jimenez D. (2019). Nat. Commun..

[cit80] Danks A. E., Hall S. R., Schnepp Z. (2016). Mater. Horiz..

[cit81] Job N., Théry A., Pirard R., Marien J., Kocon L., Rouzaud J.-N., Béguin F., Pirard J.-P. (2005). Carbon.

[cit82] Khalil K. M. S., El-Khatib R. M., Ali T. T., Mahmoud H. A., Elsamahy A. A. (2013). Powder Technol..

[cit83] Livage J., Sanchez C. (1992). J. Non-Cryst. Solids.

[cit84] Brinker C. J. and Scherer G. W., Sol–gel science: the physics and chemistry of sol–gel processing, Academic Press, Boston, 1990.

[cit85] Cravillon J., Münzer S., Lohmeier S.-J., Feldhoff A., Huber K., Wiebcke M. (2009). Chem. Mater..

[cit86] Xiang S., Li L., Zhang J., Tan X., Cui H., Shi J., Hu Y., Chen L., Su C.-Y., James S. L. (2012). J. Mater. Chem..

[cit87] Choi K. M., Jeon H. J., Kang J. K., Yaghi O. M. (2011). J. Am. Chem. Soc..

[cit88] Deville S. (2013). J. Mater. Res..

[cit89] Ahmed A., Hasell T., Clowes R., Myers P., Cooper A. I., Zhang H. (2015). Chem. Commun..

[cit90] Zhang J., Su C.-Y. (2013). Coord. Chem. Rev..

[cit91] Peng Y., Krungleviciute V., Eryazici I., Hupp J. T., Farha O. K., Yildirim T. (2013). J. Am. Chem. Soc..

[cit92] He Y., Zhou W., Qian G., Chen B. (2014). Chem. Soc. Rev..

[cit93] Yu J., Xie L.-H., Li J.-R., Ma Y., Seminario J. M., Balbuena P. B. (2017). Chem. Rev..

[cit94] Hong W. Y., Perera S. P., Burrows A. D. (2015). Microporous Mesoporous Mater..

[cit95] Hou J., Dong G., Ye Y., Chen V. (2014). J. Membr. Sci..

[cit96] Asif M. B., Hai F. I., Hou J., Price W. E., Nghiem L. D. (2017). J. Environ. Manage..

[cit97] Xia W., Zhang X., Xu L., Wang Y., Lin J., Zou R. (2013). RSC Adv..

[cit98] Ahmad R., Wong-Foy A. G., Matzger A. J. (2009). Langmuir.

[cit99] Ahmed A., Forster M., Clowes R., Myers P., Zhang H. (2014). Chem. Commun..

[cit100] Wang A., Jin M., Li N., Ma Y., Chen L., Ma D., Chen B.-X., Gao F., Tian Y.-Q., Shi Y.-K. (2017). New J. Chem..

[cit101] Mukherjee S., Kansara A. M., Saha D., Gonnade R., Mullangi D., Manna B., Desai A. V., Thorat S. H., Singh P. S., Mukherjee A., Ghosh S. K. (2016). Chem. – Eur. J..

[cit102] Jayaramulu K., Datta K. K. R., Rösler C., Petr M., Otyepka M., Zboril R., Fischer R. A. (2016). Angew. Chem., Int. Ed..

[cit103] Qin Z.-S., Dong W.-W., Zhao J., Wu Y.-P., Zhang Q., Li D.-S. (2018). Inorg. Chem. Front..

[cit104] Vermoortele F., Bueken B., Le Bars G., Van de Voorde B., Vandichel M., Houthoofd K., Vimont A., Daturi M., Waroquier M., VanVan
SpeybroeckSpeybroeck V., Kirschhock C., De Vos D. E. (2013). J. Am. Chem. Soc..

[cit105] Ramos-Fernandez E. V., Garcia-Domingos M., Juan-Alcañiz J., Gascon J., Kapteijn F. (2011). Appl. Catal., A.

[cit106] Fang Z., Bueken B., De Vos D. E., Fischer R. A. (2015). Angew. Chem., Int. Ed..

[cit107] Shalygin A. S., Nuzhdin A. L., Bukhtiyarova G. A., Martyanov O. N. (2017). J. Sol-Gel Sci. Technol..

[cit108] Chen Y., Huang X., Zhang S., Li S., Cao S., Pei X., Zhou J., Feng X., Wang B. (2016). J. Am. Chem. Soc..

[cit109] Mehta J. P., Tian T., Zeng Z., Divitini G., Connolly B. M., Midgley P. A., Tan J.-C., Fairen-Jimenez D., Wheatley A. E. H. (2018). Adv. Funct. Mater..

[cit110] Mahmood A., Zou R., Wang Q., Xia W., Tabassum H., Qiu B., Zhao R. (2016). ACS Appl. Mater. Interfaces.

[cit111] Dazon C., Witschger O., Bau S., Fierro V., Llewellyn P. L. (2019). Environ. Sci.: Nano.

[cit112] Avci C., Imaz I., Carné-Sánchez A., Pariente J. A., Tasios N., Pérez-Carvajal J., Alonso M. I., Blanco A., Dijkstra M., López C., Maspoch D. (2017). Nat. Chem..

[cit113] Carné-Sánchez A., Craig G. A., Larpent P., Hirose T., Higuchi M., Kitagawa S., Matsuda K., Urayama K., Furukawa S. (2018). Nat. Commun..

